# No evidence of *Bartonella* infections in host-seeking *Ixodes scapularis* and *Ixodes pacificus* ticks in the United States

**DOI:** 10.1186/s13071-024-06386-3

**Published:** 2024-08-19

**Authors:** Ying Bai, Kristin L. McClung, Lynn M. Osikowicz, Sarah Maes, Rebecca J. Eisen

**Affiliations:** 1 Division of Vector-Borne Diseases, National Center for Emerging and Zoonotic Infectious Diseases, Centers for Disease Control and Prevention, Fort Collins, CO USA; 2 US Department of Agriculture, National Wildlife Research Center, Wildlife Services, Animal and Plant Health Inspection Service, Fort Collins, CO USA

**Keywords:** *Bartonella* spp., *Ixodes scapularis*, *I. pacificus*, Host-seeking, Next-generation sequencing, United States

## Abstract

**Background:**

*Bartonella* spp. infect a variety of vertebrates throughout the world, with generally high prevalence. Several *Bartonella* spp. are known to cause diverse clinical manifestations in humans and have been recognized as emerging pathogens. These bacteria are mainly transmitted by blood-sucking arthropods, such as fleas and lice. The role of ticks in the transmission of *Bartonella* spp. is unclear.

**Methods:**

A recently developed quadruplex polymerase chain reaction (PCR) amplicon next-generation sequencing approach that targets *Bartonella*-specific fragments on *gltA*, *ssrA*, *rpoB*, and *groEL* was applied to test host-seeking *Ixodes scapularis* ticks (*n* = 1641; consisting of 886 nymphs and 755 adults) collected in 23 states of the eastern half of the United States and *Ixodes pacificus* ticks (*n* = 966; all nymphs) collected in California in the western United States for the presence of *Bartonella* DNA. These species were selected because they are common human biters and serve as vectors of pathogens causing the greatest number of vector-borne diseases in the United States.

**Results:**

No *Bartonella* DNA was detected in any of the ticks tested by any target.

**Conclusions:**

Owing to the lack of *Bartonella* detection in a large number of host-seeking *Ixodes* spp. ticks tested across a broad geographical region, our results strongly suggest that *I. scapularis* and *I. pacificus* are unlikely to contribute more than minimally, if at all, to the transmission of *Bartonella* spp.

**Graphical Abstract:**

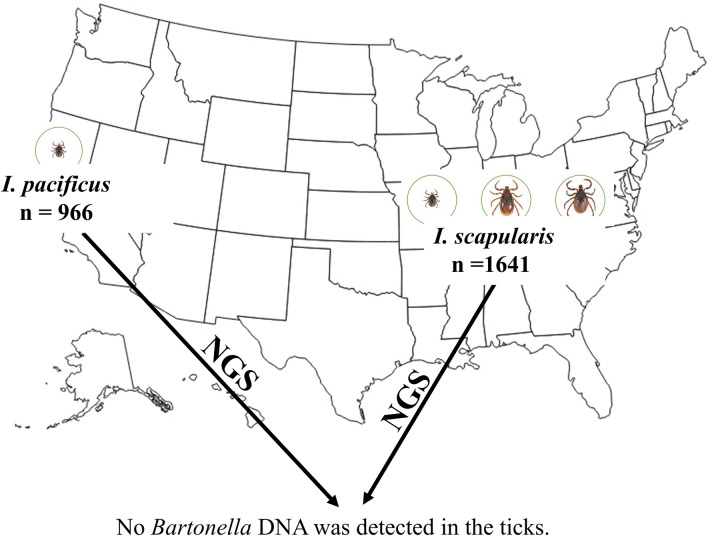

## Background

*Bartonella* is a diverse genus of gram-negative bacteria that are highly adapted to intracellular persistence in a wide range of vertebrates. Many mammalian species are natural reservoirs for *Bartonella* spp. and experience chronic, asymptomatic, intraerythrocytic bacteremia when infected [1]. Over the last two decades, more than 40 species belonging to the genus *Bartonella* have been described from different mammalian hosts, with more than a dozen having been associated with diverse clinical manifestations of diseases in humans and recognized as emerging pathogens [2–9]. Many *Bartonella* spp. are host-specific, suggesting maintenance of individual species in independent enzootic cycles [10].

*Bartonella* spp. are typically vector-borne, transmitted between mammalian hosts by hematophagous arthropods. To date, several arthropods have been proven to be vectors for *Bartonella* spp. transmission. Sand flies (*Lutzomyia verrucarum*), human body lice (*Pediculus humanis corporis*), and cat fleas (*Ctenocephalides felis*) are well-known vectors that transmit *Bartonella bacilliformis* (Carrión’s disease) [11], *B. quintana* (trench fever) [12, 13], and *B. henselae* (cat scratch disease) [14], respectively. Additionally, the Oriental rat flea (*Xenopsylla cheopis*) and other rodent fleas have been implicated as potential vectors transmitting rodent-associated *Bartonella* spp., such as *B. elizabethae*, *B. grahamii*, and *B. taylorii* [15, 16].

In the United States, the majority of reported vector-borne disease cases are caused by pathogens spread by ticks [17]. There has been considerable interest in ticks as potential vectors for *Bartonella* species. Numerous molecular surveys to detect *Bartonella* DNA in various tick species from around the world have been conducted in recent years [18–39]. Studies focused on host-seeking ticks are particularly intriguing because detection of *Bartonella* in unfed ticks would suggest that the bacterium can survive the transstadial molt from larvae to nymphs or from nymphs to adults; demonstration of transstadial transmission is one component in demonstrating vector competence [40]. The results from these studies were discordant. Some studies reported detection of *Bartonella* DNA with high prevalence in host-seeking ticks. For example, Chang et al. [19] detected *B. quintana* and several other *Bartonella* species by *gltA* in 19.2% of host-seeking *Ixodes pacificus* ticks in California, USA; Adelson et al. [21] reported that 34.5% of host-seeking *Ixodes scapularis* ticks collected in New Jersey, USA, were infected with *Bartonella* spp. using 16S primers; Morozova et al. [22] identified *B. henselae* by *groEL* in as much as 44% of host-seeking *Ixodes persulcatus* ticks from Russia. By contrast, other studies reported very low prevalence of *Bartonella* in ticks. For example, only 0.9% of host-seeking *I. scapularis* ticks from Maryland, USA [27], and 0.2% of host-seeking *I. ricinus* ticks from France [29] harbored *Bartonella* DNA; both studies were based on *gltA* results. Moreover, absence of *Bartonella* in host-seeking ticks was reported in studies from Hungary, Korea, and Finland based on the results of *gltA*, *groEL*, or *ssrA* [25, 28, 41].

With these varied results, it has been debated whether ticks can serve as vectors of *Bartonella* spp. In the USA, the blacklegged tick (*I. scapularis*) and the western blacklegged tick (*I. pacificus*) are frequent human-biters in the eastern and far western USA, respectively [42]. Both species serve as vectors of the Lyme disease spirochete, as well as several other human pathogens [43–45]. In this study, we tested host-seeking *I. scapularis* and *I. pacificus* nymphs and adults collected across 24 states for the presence of *Bartonella* DNA. Previous surveys mainly used traditional polymerase chain reaction (PCR) with a single target [18–38]. Because of the genetic diversity of *Bartonella* spp., however, a single target may not be able to detect all species, and in some cases, single targets might not be sufficiently specific to accurately detect *Bartonella*, yielding false-positive results. In our study, we applied a recently described quadruplex PCR amplicon next-generation sequencing approach which amplifies and sequences four *Bartonella*-specific gene targets (*gltA*, *ssrA*, *rpoB*, and *groEL*) [46]. The use of multiple target sequences increases detection success and also improves the accuracy of species identification compared to single-target methods. Our objective was to elucidate whether *Bartonella* DNA is present in the host-seeking *Ixodes* ticks using this powerful detection method and to infer whether the ticks can serve as vectors for transmission of *Bartonella* spp. based on the prevalence of infection in ticks collected over a broad geographical area in the USA.

## Methods

### Tick collections and DNA templates

The ticks tested in this study were from our archived DNA samples from ticks collected as part of national tick surveillance [47] or research efforts. Host-seeking *I. scapularis* were collected in 23 states in the eastern half of the USA by dragging or flagging during 2013–2023. Ticks included in this study were chosen based on life stage, sex, and collection site. We were aiming to include ~100 ticks from each state, but the actual number of ticks per state varied between 13 and 153 due to low availability in some states or inclusion of more collection sites in other states. As a result, a total of 1641 *I. scapularis* ticks consisting of 886 nymphs and 755 adults (330 males and 425 females) were tested for the presence of *Bartonella* spp. (Table 1). DNA was extracted previously using the KingFisher DNA extraction system (Thermo Fisher Scientific, Waltham, MA, USA) and the MagMAX™ CORE Kit (Thermo Fisher Scientific) [48] for tick surveillance testing and stored at −80 °C afterwards. Residual DNA was used for the present study.

**Table 1:** Host-seeking *I. scapularis* ticks collected in 23 eastern US state during 2013–2023 for *Bartonella* DNA presence testing

Region^a^/state	No. of ticks tested	No. of nymphs tested	No. of adults tested		Year(s) collected	*Bartonella* DNA presence			
			Female	Male		*gltA*	*ssrA*	*rpoB*	*groEL*
Northeast									
Connecticut	45	45			2023	Neg	Neg	Neg	Neg
Maine	57	57			2015	Neg	Neg	Neg	Neg
Maryland	36	36			2014	Neg	Neg	Neg	Neg
New York	96	96			2014–2015	Neg	Neg	Neg	Neg
Pennsylvania	59	59			2014–2015	Neg	Neg	Neg	Neg
Vermont	104	9	95		2018	Neg	Neg	Neg	Neg
New Hampshire	45	38	7		2022	Neg	Neg	Neg	Neg
Northern Rockies and Plains									
Nebraska	78	1	33	44	2021–2022	Neg	Neg	Neg	Neg
Ohio Valley									
Indiana	88	88			2017–2019	Neg	Neg	Neg	Neg
Tennessee	35		23	12	2018–2019	Neg	Neg	Neg	Neg
West Virginia	98	98			2020–2022	Neg	Neg	Neg	Neg
Kentucky	13	13			2019	Neg	Neg	Neg	Neg
Ohio	104		75	29	2019, 2021–2022	Neg	Neg	Neg	Neg
Southeast									
North Carolina	121	50	28	43	2016–2019, 2022–2023	Neg	Neg	Neg	Neg
Virginia	97	75	7	15	2013–2015, 2018–2019	Neg	Neg	Neg	Neg
Alabama	33	5	20	8	2017	Neg	Neg	Neg	Neg
South Carolina	53	3	26	24	2020–2021	Neg	Neg	Neg	Neg
South									
Mississippi	49	49			2013, 2015–2016	Neg	Neg	Neg	Neg
Oklahoma	60		32	28	2021	Neg	Neg	Neg	Neg
Upper Midwest									
Iowa	153	56	36	61	2019–2021	Neg	Neg	Neg	Neg
Michigan	74	17	22	35	2016–2018	Neg	Neg	Neg	Neg
Minnesota	43	43			2015–2016, 2022	Neg	Neg	Neg	Neg
Wisconsin	100	48	21	31	2018–2019	Neg	Neg	Neg	Neg
Total	1641	886	425	330	2013–2023	Neg	Neg	Neg	Neg

^a^Climate regions of the eastern half of the United States, as defined by the National Oceanic and Atmospheric Administration (NOAA, 2023)

Host-seeking *I. pacificus* ticks were collected from different woodland habitat types in highly climatically and ecologically diverse Mendocino County, CA, by dragging in 2004 as previously described [49]. A total of 966 *I. pacificus* nymphs with representatives of all woodland habitat types described in the county were tested for the presence of *Bartonella* spp. (Table 2). Total DNA had been extracted from individual nymphs previously using the DNeasy Blood & Tissue Kit (Qiagen, Valencia, CA, USA) [50], and archived DNA was used for the present study.

**Table 2:** Host-seeking *I. pacificus* nymphal ticks collected from different woodland habitat types in Mendocino County, CA, 2004, for *Bartonella* DNA presence testing

Habitat type	No. of nymphs tested	*Bartonella* DNA presence			
		*gltA*	*ssrA*	*rpoB*	*groEL*
Redwood	74	Neg	Neg	Neg	Neg
Coastal pine	13	Neg	Neg	Neg	Neg
Inland pine	149	Neg	Neg	Neg	Neg
Oak–madrone (hardwood)	278	Neg	Neg	Neg	Neg
Hardwood–conifer	305	Neg	Neg	Neg	Neg
Tanoak	107	Neg	Neg	Neg	Neg
Tanoak–madrone–conifer (mixed class)	40	Neg	Neg	Neg	Neg
Total	966	Neg	Neg	Neg	Neg

### PCR amplification, library preparation, and next-generation sequencing

Ticks were tested for the presence of *Bartonella* DNA using a quadruplex PCR amplicon next-generation sequencing assay that targets *gltA*, *ssrA*, *rpoB*, and *groEL* using *Bartonella*-specific primers [46]. Additionally, an internal control using 16S ribosomal RNA (rRNA) primer (forward 5′-CTGCTCAATGATTTTTTAAATTGCTGTGG-3′ and reverse 5′-CCGGTCTGAACTCAGATCAAGT-3′) [51] was included to ensure the presence of tick DNA and to confirm the original morphological identification of tick species.

Detailed procedures follow those described in Bai et al. [46]. Briefly, a primary PCR reaction containing 12.5 μl of TEMPase 2× master mix (AMPLICON, Denmark), the four pairs of *Bartonella*-specific primers and the 16S rRNA tick-specific primer (final concentration of 300 nM each), and 5 μl of tick DNA was first amplified. Positive and negative controls were always included in each PCR run to evaluate performance and detect contamination. Upon completion of amplification, the PCR products were purified with AMPure XP magnetic beads (Beckman Coulter, Brea, CA, USA), followed by index PCR using dual unique barcode indices (Nextera XT Index Kit V2, Illumina, San Diego, CA, USA), then purified with MagSi-DNA allround magnetic beads (BOCA Scientific, Westwood, MA, USA). The purified products were then pooled, quantified, normalized, and denatured to generate the final library to be loaded into a MiSeq Nano v2 (500 cycles) reagent cassette (Illumina, San Diego, CA, USA) to start sequencing on an Illumina MiSeq instrument (Illumina, San Diego, CA, USA).

### Tick DNA interference testing

The inhibitory effect is a commonly observed issue during PCR amplification, which sometimes causes false-negative results for pathogen detection [52]. Before applying the assay for field tick testing, we evaluated the assay performance to check whether tick DNA would cause any inhibition or interference during PCR amplification. Clean tick DNA was extracted from *I. scapularis* ticks raised at the Centers for Disease Control and Prevention (CDC) colony using the KingFisher DNA extraction system; *Bartonella* DNA from pure culture of *B. henselae*, *B. koehlerae*, and *B. grahamii* (collections at CDC Division of Bacterial Diseases branch in Fort Collins, CO, USA) was prepared by heating a heavy suspension of microorganisms for 15 min at 95 °C followed by centrifugation of the lysed cells for 1 min at 8000 rpm. The supernatant was transferred to a clean centrifuge tube to be used as the DNA template.

The DNA concentration was measured using the Invitrogen™ Qubit™ 4 Fluorometer dsDNA [double-stranded DNA] HS Assay (Fisher Scientific, Pittsburgh, PA, USA). Then a high concentration of the clean tick DNA (5 ng/μl) was mixed with DNA of *B. henselae* (10 pg/μl), *B. koehlerae* (10 pg/μl), and *B. grahamii* (10 pg/μl), respectively. Five microliters of each mixture was then used for the interference testing following the procedures described in the preceding section. Triplicates of each mixture were tested.

### Bioinformatics analysis

After sequencing was completed, the raw sequences were analyzed with a custom Nextflow bioinformatics pipeline described by Osikowicz et al. [53]. Briefly, quality control analysis and primer trimming were first performed followed by error correction, paired read merging, and amplicon sequence variant (ASV) grouping. The observed ASVs were then aligned to reference sequences with the nucleotide Basic Local Alignment Search Tool (BLASTn) [54, 55]. The minimum read cut-off for a sample to be considered positive was set to 50 reads. A 95% sequence similarity and 90% minimum sequence alignment length were used to align the observed ASVs to the reference sequences. Sequences that represent different *Bartonella* species for each target were obtained from GenBank and used as reference sequences.

## Results

### Tick DNA interference testing

Spiked DNA from *B. henselae*, *B. koehlerae*, and *B. grahamii* was successfully detected and identified by each of the four targets used in the quadruplex sequencing in all triplicates of each mixture of tick DNA and *Bartonella* DNA, showing no interference from tick DNA.

### Host-seeking tick testing

The internal control with the 16S rRNA tick primer showed that tick DNA was present in all samples. All ticks from the eastern USA were confirmed to be *I. scapularis* ticks, and all ticks from Mendocino County, CA, were confirmed to be *I. pacificus*.

No *Bartonella* DNA was detected in any ticks, nymph or adult, by any target (Tables 1 and 2).

## Discussion

*Ixodes scapularis* and *I. pacificus* ticks are important vectors in the USA that are responsible for transmission of *Borrelia burgdorferi* (the Lyme disease agent) and several other human pathogens [43–45]. However, the role of these ticks in *Bartonella* transmission was not clear. In the present study, we tested more than 2600 host-seeking *I. scapularis* and *I. pacificus* ticks for the presence of *Bartonella* DNA using a sensitive and specific quadruplex PCR amplicon sequencing approach. No *Bartonella* DNA was detected in any ticks, nymphs, or adults, by any target. Similar results have been reported by other investigators who tested host-seeking *Ixodes* ticks across more limited spatial scales but found no detectable *Bartonella* DNA [25, 28, 41]. These results demonstrate that host-seeking *Ixodes* ticks are unlikely to contribute to the transmission of *Bartonella* spp.

Previous studies that tested blood-fed *Ixodes* ticks collected from different animals were able to detect *Bartonella* infections [56, 57], demonstrating that *Ixodes* ticks are exposed to *Bartonella* through blood-feeding on infected hosts. *Bartonella* infections are prevalent in rodent species that commonly serve as blood meal sources for *I. scapularis* and *I. pacificus* [58, 59]. However, the lack of detection of *Bartonella* in unfed *Ixodes* ticks, which take only a single blood meal per life stage, suggests that the bacteria seldom, if ever, survive the transstadial molt from larva to nymph or nymph to adult. The lack of *Bartonella* DNA in adult ticks is particularly compelling to support the conclusion that *Bartonella* does not survive the transstadial molt, because adult ticks could have been exposed to any potential infections twice by taking two blood meals.

*Bartonella* spp. typically cause long-lasting hemotrophic bacteremia in their mammalian reservoir hosts. The reservoir hosts, however, may clear the infection after being bacteremic for several months, but later may acquire the infection again, with the same or a different strain [58, 60]. The temporal dynamics observed in mammals may apply to the ticks as well. Both *I. scapularis* and *I. pacificus* ticks go through four life stages (egg, larva, nymph, and adult) to complete their life cycles. After a blood meal, the ticks take months to a year to digest and molt into the next life stage. During the long molting process, *Bartonella* infection might have been cleared, assuming the ticks were infected during the previous life stage. This may explain the absence of *Bartonella* DNA in host-seeking ticks. Alternatively, *Bartonella* could have deleterious effects on tick survival, resulting in very low pathogen prevalence in surviving host-seeking ticks.

Notably, molecular detection of *Bartonella* DNA has been reported in ticks (primarily *Ixodes* spp.) collected at various locations in the USA, Europe, and other parts of the world, with high or low infection rates. It is worth noting that high *Bartonella* prevalence in ticks (> 30%) were mostly from studies using 16S rRNA [21, 30]. Such results are questionable due to the highly conserved 16S rRNA target used, which had little genetic diversity and shared homology with non-target microbes [61, 62]. Studies utilizing non-specific targets could yield a falsely high prevalence of *Bartonella* infections in ticks. On the other hand, lower *Bartonella* prevalence in host-seeking *Ixodes* ticks (< 1%) has been reported, mostly in studies using much more specific genes, for example, *gltA* [27, 29, 37]. Such low prevalence is consistent with our findings. Although we tested over 2600 ticks in our study, we cannot rule out the possibility that *Bartonella* infections could occur at very low prevalence in host-seeking *Ixodes* spp. ticks. However, it is important to emphasize that the mere presence of *Bartonella* DNA within a tick does not imply that the bacteria are viable or that the tick could transmit it during the course of blood-feeding [61].

Although our field investigation suggests that *I. scapularis* and *I. pacificus* are unlikely to contribute to transmission of *Bartonella* spp., laboratory experiments demonstrated the vector competence of other *Ixodes* ticks, in particular *I. ricinus*, which could acquire *B. henselae* and *B. birtlesii* through blood-feeding, maintain the infections throughout the molt, and transmit *B. henselae*/*B. birtlesii* during a subsequent blood meal [63, 64]. *Bartonella* is a very diverse taxon, and it is possible that vector competence and efficiency differ across *Ixodes* and *Bartonella* species combinations. However, in those studies, the *I. ricinus* ticks were continuously fed on blood with a very high bacteremia load (10^8^–10^9^ CFU) [63, 64], which is rarely seen in natural infections. Thus, the experimental results may not be relevant to establishing vector competence under natural conditions. Nevertheless, vector competence has not been demonstrated for *I. scapularis* or *I. pacificus* and any *Bartonella* species that naturally occur in the USA. Laboratory transmission studies on *I. scapularis* and *I. pacificus* are needed to elucidate acquisition, survival, and transmission rates.

## Conclusions

Although sample sizes were low for many states, our testing spanned a broad geographical region, and cumulatively a large number of ticks were tested. Using a highly sensitive and specific next-generation sequencing approach, we tested more than 2600 host-seeking *I. scapularis* and *I. pacificus* ticks from 24 states. No *Bartonella* DNA was detected in the ticks. Our data, together with previous studies from more limited geographical regions [27, 38, 39], strongly suggest that *I. scapularis* and *I. pacificus* are unlikely to contribute more than minimally, if at all, to the transmission of *Bartonella* spp. in the USA.

## Data Availability

All data of this study are presented in the manuscript.
